# Synthesis, Characterization, and Anti-diabetic Activity of Some Novel Vanadium-Folate-Amino Acid Materials

**DOI:** 10.3390/biom10050781

**Published:** 2020-05-18

**Authors:** Ahmed M. Naglah, Mohamed A. Al-Omar, Abdulrahman A. Almehizia, Ahmad J. Obaidullah, Mashooq A. Bhat, Atef Kalmouch, Asma S. Al-Wasidi, Jehan Y. Al-Humaidi, Moamen S. Refat

**Affiliations:** 1 Department of Pharmaceutical Chemistry, Drug Exploration & Development Chair (DEDC), College of Pharmacy, King Saud University, Riyadh 11451, Saudi Arabia; malomar1@ksu.edu.sa (M.A.A.-O.); mehiza@ksu.edu.sa (A.A.A.); 2 Peptide Chemistry Department, Chemical Industries Research Division, National Research Centre, 12622 Dokki, Cairo, Egypt; atefkalmush@gmail.com; 3 Department of Pharmaceutical Chemistry, College of Pharmacy, King Saud University, Riyadh 11451, Saudi Arabia; aobaidulah@ksu.edu.sa (A.J.O.); mabhat@ksu.edu.sa (M.A.B.); 4 Department of Chemistry, College of Science, Princess Nourah Bint Abdulrahman University, Riyadh 11671, Saudi Arabia; asmachem7@hotmail.com (A.S.A.-W.); jyhumaidi@gmail.com (J.Y.A.-H.); 5 Department of Chemistry, Faculty of Science, Taif University, P.O. Box 888, Al-Hawiah, Taif 21974, Saudi Arabia; msrefat@yahoo.com; 6 Department of Chemistry, Faculty of Science, Port Said University, 42526 Port Said, Egypt

**Keywords:** insulin alternative, diabetes, drug, VO^2+^ ion, folic acid, amino acid, spectroscopic

## Abstract

A new six intraperitoneal injections insulin-mimetic vanadyl(IV) compounds [(VO)(FA)(AA_n_)] (where n = 1–6: AA_1_ = isoleucine, AA_2_ = threonine, AA_3_ = proline, AA_4_ = phenylalanine, AA_5_ = lysine, and AA_6_ = glutamine) were synthesized by the chemical reactions between folic acid (FA), VOSO_4_, and amino acids (AA_n_) with equal molar ratio 1:1:1 in neutralized media. These complexes were characterized by elemental analysis and estimation of vanadyl(IV) metal ions. The thermal stability behavior of these complexes was studied by TG-DTG-DTA analyses. The structures of these complexes were elucidated by spectroscopic methods like infrared, electron spin resonance (ESR), and solid reflectance spectroscopes. The powder X-ray diffraction (XRD) study suggested the crystalline nature of the complexes. Magnetic moments and electronic spectra revealed the square-pyramid geometrical structure of the complexes. The conductivity results refereed that all synthesized vanadyl(IV) complexes were of a non-electrolyte behavior. The infrared spectra assignments of these complexes revealed that the FAH_2_ and AA_n_ chelates act as a bidentate ligation. The chelation towards vanadyl (IV) ions existed via deprotonation of one of the carboxylic groups of FAH_2_ drug ligand, and so amino acids act as bidentate ligands via N-amino and O-carboxylate groups. Both scanning and transmission electron microscope (SEM and TEM) techniques were used to investigate the surface morphology. The main task of this research is the aim of designing a new insulin alternative antidiabetic drug agent. The antidiabetic efficiency of these complexes was evaluated in streptozotocin-induced diabetic male albino rats. Liver and kidney functions, insulin and blood glucose levels, lipid profile, and superoxide dismutase antioxidant (SOD) are verified identifiers for the efficiency of VO(IV)/FA/AA_n_ system compounds as antidiabetic drug agents.

## 1. Introduction

The increasing knowledge of the role of vanadium in biological systems and the potential of vanadium compounds as therapeutic agents have led to increased interest in the chemistry of coordination [1,2,3,4]. Oxovanadium(V) compounds have been utilized as a model of insulin-mimetic therapeutic agents [5,6]. Moreover, there are several medical activities regarding vanadium compounds [4,5,6,7]. Diabetes is the main causative of numerous kidney diseases as well as heart attacks that may result in patient’s blindness and amputation of limbs [8,9]. The insufficiency of the pancreas’ beta cells is the main cause of path physiological markers in the progression of diabetes 1 or 2 [10,11,12].

Folic acid (FAH_2_) is commonly named as vitamin B_9_ [13]; it was initially isolated from a spinach plant’s leaves [14] and then used to remedy in anemia of a megaloblastic nature. Mainly, folic acid is a vitamin that is water-soluble and plays a vital role in a variety of humans’ physiological functions. It played a crucial role in one-carbon metabolism for physiological DNA synthesis and cell division [15,16,17,18,19]. Nowadays, it is known that deficiency of folic acid is associated with higher plasma concentrations of homocysteine, a self-reliant risk factor for birth defects and pregnancy complications [20]. Folic acid crucially contributes to the prevention of neural tube defects in infants, not to mention its inhibition of vascular diseases and certain cancers [21].

As basic components of living organisms, amino acids take part in proteins’ block building, which are chemical species indispensable to performing a large number of biological functions [22]. Amino acids and their mixed ligand complexes are found in applications of biology, industry, pharmacy, and laboratory reagents [23], with involvement in the human body various activities, e.g., transamination, pH regulation, neurotransmitter functions, cholesterol metabolism, inflammation control, decarboxylation, pain administration, and detoxification.

This paper focuses on the preparation, spectroscopic, and biological characterizations of the new vanadyl(IV) folate amino acid series complexes for the utility of treatment of diabetes that was induced by streptozotocin (STZ) in male albino general rats. 

## 2. Materials and Methods 

### 2.1. Chemical and Reagents

In this study, the used chemicals and reagents were of the highest purity (Aldrich, Saint Louis, MO, USA) and without any further purifications. The folic acid pure drug, vanadyl(IV) sulfate, and amino acids (isoleucine, threonine, proline, phenylalanine, lysine, and glutamine) were bought from the Aldrich Company. 

### 2.2. Synthesis of Vanadyl(IV)–Folate–Amino Acid Complexes

For all preparations, a solvent of doubly distilled water was employed. The solid vanadyl(IV) –folate–amino acid complexes with general formula NH_4_[(VO)(FA)(AA_n_)] (I-VI) (where AA_1_ = isoleucine, AA_2_ = threonine, AA_3_ = proline, AA_4_ = phenylalanine, AA_5_ = lysine, and AA_6_ = glutamine) were prepared by employing equal molar ratios of (FAH_2_:VOSO_4_:AA_n_). These complexes were isolated by directly mixing 1.0 mmol of isoleucine (0.065 g), threonine (0.059 g), proline (0.057 g), phenylalanine (0.082 g), lysine (0.073 g), and glutamine (0.073 g) with 40 cm^3^ of CH_3_OH/H_2_O solution of folic acid (0.22 g, 1.0 mmol), and then VOSO_4_.H_2_O salt (0.09 g, 1.0 mmol) was added. All these mixtures were neutralized using diluted ammonia solution (conc. 5%) at pH (8–9) and refluxed at ~60 °C for 30 min till the precipitates settled down, then they were filtered off, washed three times using few amounts of warming methanol, dried at 60 °C, and then stored in a desiccator over anhydrous CaCl_2_.

### 2.3. Instruments

A Perkin Elmer CHN 2400 (PerkinElmer Inc., Shelton, CT, USA) was used to conduct the analysis of %C, %H and %N element content. Vanadium content was determined gravimetrically as V_2_O_5_. A Jenway 4010 conductivity meter (Jenway, Staffordshire, UK) was used for the molar conductivity measurements of the samples at 10^−3^ M in dimethylsulfoxide (DMSO). A 3101 PC UV/Vis spectrophotometer (Shimadzu Scientific Instruments, Kyoto, Japan) was used to scan the solid refelactance spectra for samples. A Bruker FT−IR spectrophotometer (Bruker, Billerica, MA, USA) was used to collect the IR spectra for solid samples on KBr discs within the 4000–400 cm^−1^ range. A Shimadzu TG/DTG–50H thermogravimetric analyzer (Shimadzu Scientific Instruments) was employed for the thermal analyses of solid samples under atmospheric nitrogen conditions. A X’Pert Philips X-ray diffractometer (Philips, Manchester, UK) was used to collect the X-ray diffraction pattern (XRD) patterns for the solid samples using CuK_α1_ radiation. Scanning electron microscopy (SEM) and transmission electron microscopy (TEM) micrographs were obtained using a Jeol Jem−1200 EX II electron microscope (Jeol Ltd., Akishima, Japan) operated at an acceleration voltage of 25 kV. A Gouy magnetic balance (Sherwood Scientific Ltd., Cambridge, UK) was used to measure the mass susceptibility (X_g_) of the complexes at room temperature. Electron spin resonance was measured by Jeol, JES-FE2XG, ESR-spectrometer, Frequency 9.44 GHz (Jeol Ltd., Akishima, Japan).

### 2.4. Animal Experiments

The biological experiments were performed on male albino rats of 0.1–0.120 kg weight. These rats were received from the National Research Centre in Cairo (Egypt). These experiments were applied based on the European Community Directive (86/609/EEC) and national rules on animal care. Male rats were classified into four groups. Each group included ten rats, while Group IV included ten rats for each system.

Group I is a normal control group; Group II is a Positive control, injected intraperitoneally by a single dose of STZ (50 mg/kg body weight) [24]; Group III is a Injected i.p. by STZ, then injected each alternative day by VOSO_4_ alone with a dose of 40 mg/kg body weight through 30 days and Group IV is a Injected with STZ, and then injected each alternative day by synthesized vanadyl(IV) complexes (I–VI) with a dose of 40 mg/kg body weight [25] for 30 days.

### 2.5. Experimental Diabetes Induction

Induction of experimental diabetes was induced in rats fasting for 18 h via a single intraperitoneally (i.p.) injection of STZ with a 50 mg/kg dose [25] freshly prepared in a cold 0.1 M citrate buffer (pH 4.5). Streptozotocin -injected rats were made to drink a 5% glucose drinking solution during the initial 24 h for survival assurance [26]. Animals were considered diabetic when their blood glucose level surpassed 220 mg/dL [27]. Afterwards, they were included in the study after 72 h of STZ injection.

### 2.6. Blood and Organ Collections

Blood samples of the fasting rats were collected from the medial retro-orbital venous plexus via capillary tubes (Micro Haematocrit Capillaries, (Mucaps, Fisher Scientific, Arendalsvägen, Göteborg, Sweden)) under ether anesthesia [28]. 

### 2.7. Hematological Parameters

Hemoglobin (Hb) measurements were determined using a cell counter (Sysmex, model KX21N, Sawgrass Drive Bellport, NY, USA)) in grams per deciliter (g/dL) of blood. 

### 2.8. Insulin and Blood Glucose Levels

Insulin analysis took place via the insulin–I125 kit, according to Woodhead et al. [29], employing the use of a radioimmunoassay kit from Radio Assay System Laboratories Inc (Carson, CA, USA).

### 2.9. Lipid Profile

Triglycerides, cholesterol, high-density lipoprotein-cholesterol (HDL-c), and low-density lipoprotein-cholesterol (LDL-c) levels were gauged using a fully auto-chemistry analyzer (Roch Integra 400 plus analyzer, (Sawgrass Drive Bellport, NY, USA).

### 2.10. Liver and Kidney Functions

The level of LDH, ALT, uric acid, and creatinine were analyzed using the aforementioned fully auto-chemistry analyzer (Roch Integra 400 plus analyzer). The activities of G6PDH (glucose-6-phosphate dehydrogenase) were determined using commercial kits.

### 2.11. Determination of Blood SOD (Superoxide Dismutase)

Superoxide Dismutase was analyzed via a biodiagnostic kit. The percentage of inhibition was found to be at 560 nm as per the following equation:

(1) Inhibition Percentage = ΔA control - ΔA sample / ΔA control × 100

### 2.12. Histopathological Examination

Small pieces of liver and pancreas tissues were freshly collected directly following dissection, and then they were directly transferred to a 10% formalin solution for fixture [30].

### 2.13. Statistical Analysis 

Data were analyzed using the computer program SPSS version 15.0. Duncan’s multiple range test (*p* < 0.05) (IBM, Armonk, NY, USA) was used in accordance with Snedecor and Cochran to gauge the effect on different treated groups [31].

## 3. Results and Discussion

### 3.1. Interpretations of the Chemical Structure

The dark yellow synthesized solid vanadyl(IV)-folate-amino acid complexes were stable and soluble in dimethylsulfoxide (DMSO) and dimethylformamide (DMF) solvents after being warmed gently. The physical and analytical data revealed 1:1:1 stoichiometry between VO(IV): FAH_2_: AA_n_ (where n = 1-6: AA_1_ = isoleucine, AA_2_ = threonine, AA_3_ = proline, AA_4_ = phenylalanine, AA_5_ = lysine, and AA_6_ = glutamine). The magnetic moment data (1.70–2.20 Bohar Magneton (BM)) of six vanadyl(IV) folate amino acid complexes were assigned to be square pyramid geometry. 

#### 3.1.1. Microanalytical and Physical Data

The new six vanadyl(IV) complexes, which were synthesized in situ mixed ligands of folate and amino acid chelates, had higher melting points >260 °C with yields 80–85%. The microanalytical, physical, and chemical formulas of the synthesized complexes are listed in Table 1. The resulting data is in good agreement with the general formula of NH_4_[(VO)(FA)(AA_n_)] complexes. It was confirmed that SO_4_ ions were not present using a 10% stock solution of BaCl_2_.2H_2_O reagent. The molar conductance data of the vanadyl(IV) folate amino acid complexes dissolved in a DMSO solvent with a concentration of 10^−3^ M were found to be within the limit of 13–24 Ω^−1^·cm^2^·mol^−1^ at room temperature, confirming the non-electrolytic state [32,33,34]; hence, the molar conductance values indicated the absence of SO_4_ ions inside the coordination sphere. The experimental results were in agreement with the checkout of SO_4_ ions using BaCl_2_·2H_2_O reagent after the dissociation of vanadyl(IV) complexes in concentrated nitric acid. 

#### 3.1.2. Electronic, Magnetic, and Electron Spin Resonance Measurements

**Figure 1:**
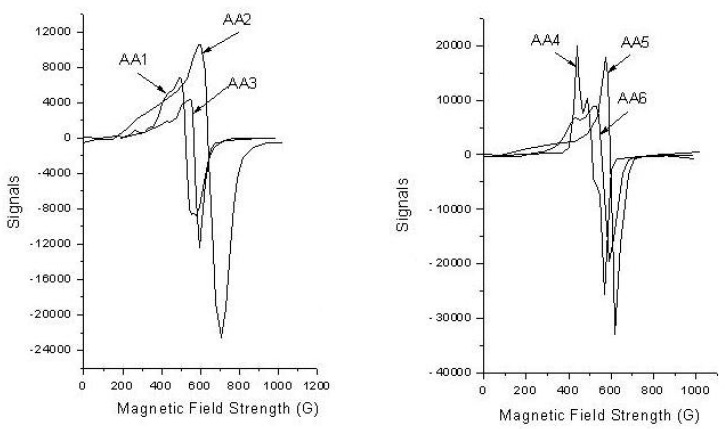
Electron spin resonance (ESR) spectra of NH_4_[(VO)(FA)(AA_n_)] complexes (where AA_1_ = isoleucine, AA_2_ = threonine, AA_3_ = proline, AA_4_ = phenylalanine, AA_5_ = lysine, and AA_6_ = glutamine) in solid-state.

The electronic diffuse reflectance spectra of NH_4_[(VO)(FA)(AA_n_)] complexes have two distinguishing bands at ranges of 12,500–12,658 cm^−1^ and 15,625–16,000 cm^−1^, which are assigned to ^2^B_2_→^2^E and ^2^B_2_→^2^B_1_ electronic transitions [35]. The absorption band presence at ranges of 17,857–18,691 cm^−1^ and 21,739–24,390 cm^−1^ are attributed to the ligand-to-metal charge transfer (L-M_CT_) band. At room temperature, the effective magnetic moment *μ_eff_* values of vanadyl(IV) folate amino acid complexes are located within 1.70–2.20 BM range; these results revealed that the geometric structure of oxovanadium(IV) complexes is square pyramid [36]. Electron Spin Resonance spectrum of the vanadyl(IV) folate amino acid complexes was measured in solid-state (Figure 1). After calculation of *g*_||_ (parallel Landé g-factor), g_┴_ (perpendicular Landé g-factor), *A*_||_ (parallel hyperfine constant), and *A*_┴_ (perpendicular hyperfine constant) items from the ESR spectra, it was found that g_||_ < g_┴_ < 2; these results agree with the square pyramid structure assumption [37].

#### 3.1.3. Infrared Spectra

**Figure 2:**
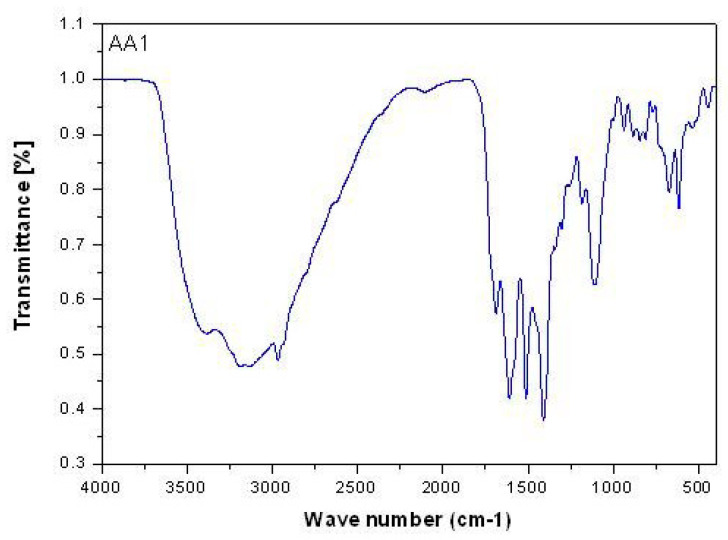
Infrared spectrum of NH_4_[(VO)(FA)(AA_1_)] complex (where AA_1_ = isoleucine).

FAH_2_, amino acids, and their mixed vanadyl(IV)complexes’ infrared spectra (Figure 2) are illustrated in Table 2.

In the case of free FAH_2_, the stretching vibration band at 1694 cm^−1^ with very strong absorption is assigned to ν(C=O)_ketonic_ of the carboxylic group [38]. However, this band is a little shifted to lower frequencies (1686–1688 cm^−1^) in the case of synthesized complexes’ spectra due to the overlapping between C=O_amid_ and C=O_carboxylic_[39,40,41,42].

There are two new bands at 1512–1483 cm^−1^ and 1410–1407 cm^−1^ regions due to ν_as_(COO^−^) and ν_s_(COO^−^) vibration motions [43].

The difference between the two vibration motions of carboxylate group Δν = ν_as_(COO-)-ν_s_(COO-)] for the vanadyl(IV) folate amino acid complexes are located within 103–74 cm^−1^ range; this value can be assigned to the association of bidentate-coordinated bond concerning the carboxylate group [44,45].

The δ(NH_2_) sharp bending vibration motion regarding FAH_2_ existsat 1607 cm^−1^; this band exists at the same frequencies in the case of synthesized vanadyl(IV) complexes. This can be attributed to the fact that the nitrogen of the NH_2_ group is far away from the coordination process.

Regarding oxovanadium(IV) folate amino acid complexes, the ν(V=O) stretching vibration motion is present as a new band at 1114–1106 cm^−1^ range [46].

NH_3_ to NH_2_ transformation must create an upward shift in ν(NH_2_) and free amino acids. In the current complexes, the infrared (IR) spectra show characteristic broad and split bands in the region 3187–3400 cm^−1^ are shown in the IR spectra, somewhat lower considering those of free ν(NH_2_); therefore, it is fair to conclude that the nitrogen of the amino group is of a pivotal role in coordination [46].

Strong evidence is imposed by the IR spectra supporting the assumption of the coordinative role of the carboxylate group. Comparing with free amino acids, the ν_as_(COO^–^) and ν_s_(COO^–^) show negative shifts, confirming the carboxylate group’s monodentate nature [46].

Hence, amino acids are concluded to act as a bidentate ligand in these complexes and coordinate through amino nitrogen and carboxylate oxygen. In the far IR spectra of all complexes, new bands viewed at 513–617 cm^−1^ and 440–460 cm^−1^ regions may be assigned for the ν(M-O) and ν(M-N), respectively [46].

**Figure 3:**
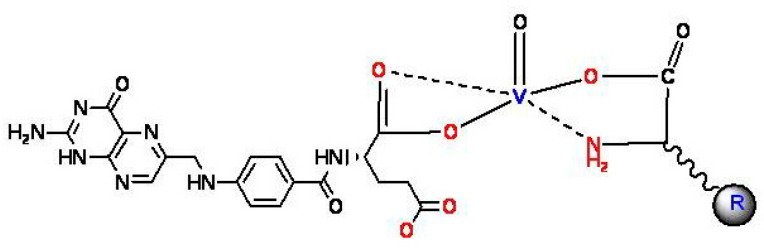
Suggested structures of vanadyl(IV) complexes (R = complementary of amino acids).

The microanalytical and spectroscopic discussions of the vanadyl(IV) folate amino acid complexes confirm the suggested stoichiometric formulations (Figure 3).

#### 3.1.4. Thermal Analysis Study

**Figure 4:**
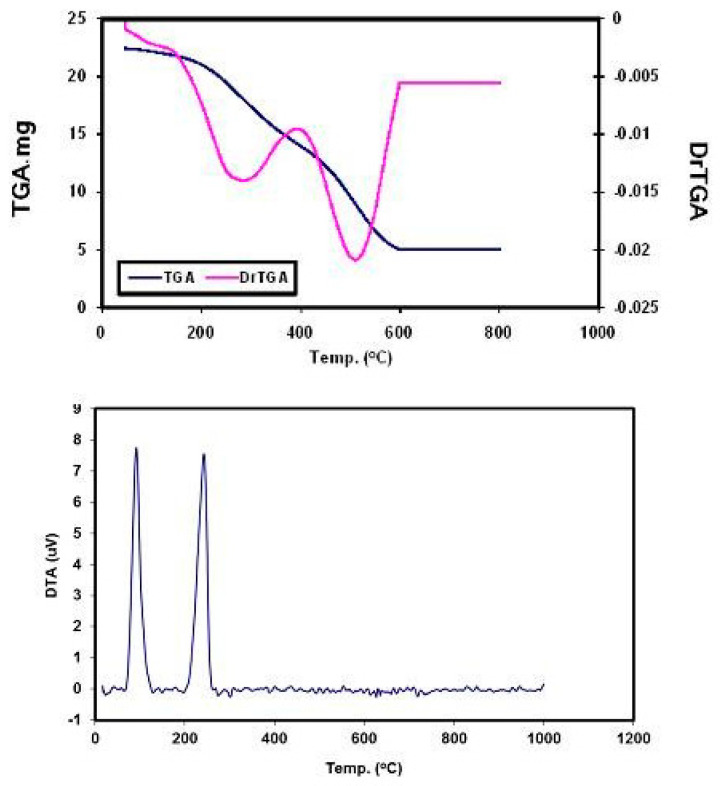
Thermogravimetric/differential thermogravimetric/differential thermal analysis (TG/DTG/DTA) curves of NH_4_[(VO)(FA)(AA_1_)] complex.

Thermal stabilities of the NH_4_[(VO)(FA)(AA_n_)] complexes were investigated based on the thermogravimetric (TG), differential thermogravimetric (DTG), and differential thermal analysis (DTA) from 25–800 °C under nitrogen atmospheres. The thermal decomposition curves are displayed in Figure 4, and the assignments of the thermal decomposition results show the thermal stability of the synthesized material. 

#### 3.1.5. X-ray powder diffraction and Transmission Electron Microscopy Studies

**Figure 5:**
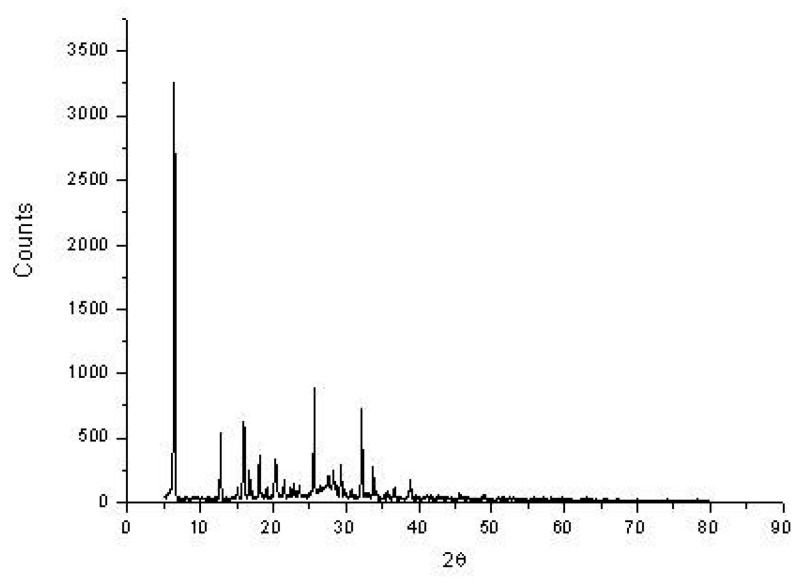
X-ray diffraction (XRD) of the solid NH_4_[(VO)(FA)(AA_1_)] complex.

**Figure 6:**
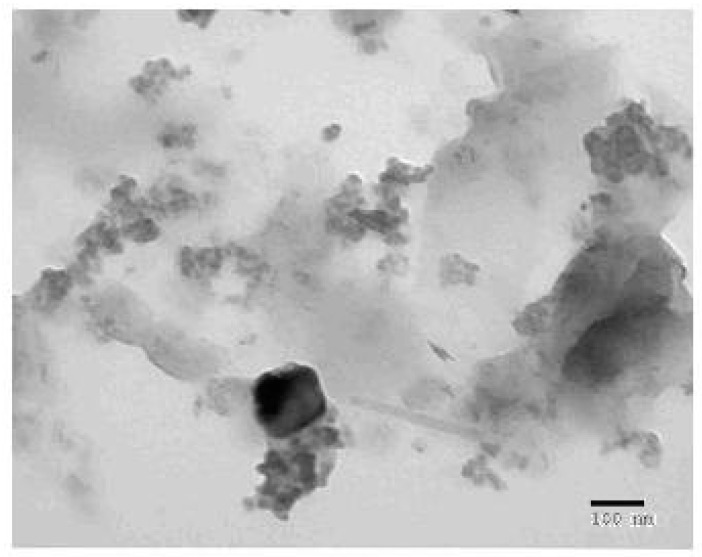
Transmission electron microscope (TEM) micrographs of the solid NH_4_[(VO)(FA)(AA_1_)] complex.

The new nanostructured form of NH_4_[(VO)(FA)(AA_1_)] complex was investigated using solid X-ray powder diffraction patterns within a 4–80° range of diffraction angle (2θ) and transmission electron microscopy (TEM). The X-ray powder diffraction (XRD) patterns deduced that the vanadyl(IV) folate complex (I) has a crystalline feature. The XRD diffraction patterns of the vanadyl(IV) complex (I) (Figure 5 and Table 3) show the presence of the characteristic peaks for vanadium 38.612° in accordance with JCPDS File 22-1058 [47], folic acid (14.996, 16.870, 18.185, 20.620, 21.548, 22.862, 25.664, 28.293, and 36.738°) [48], and isoleucine (12.735, 19.113, 32.235, and 35.791°). Vanadyl(IV) folate complex crystallite size can be gauged via the Sherrer formula (Equation (2)) [49]:

(2) D = 0.89λ / βCosθ

where Dis the particle size, 0.89 is the Sherrer constant, λ represents the wavelength of the radiation of the X-ray (0.154056 nm for Cu Kα), and β stands for the full-width half-maximum (FWHM) of diffraction peak measured at 2*θ*. The calculation of the particle size of the NH_4_[(VO)(FA)(AA_1_)] complex from the highest line diffraction peak at 6.377°. The XRD pattern of vanadyl(IV) folate complex (I) has a nanocrystalline statement with a 5 nm size. According to the TEM image (Figure 6), the particles of NH_4_[(VO)(FA)(AA_1_)] complex exhibit irregular black stones forms, and their size is widely distributed between 50–100 nm. 

### 3.2. Biological Investigation of Vanadyl(IV) Folate Amino Acids (AA_1_–AA_6_) Complexes on Diabetic Rats

#### 3.2.1. Blood Glucose and Insulin Hormone Levels

Blood glucose and insulin hormone levels in the experimental groups are illustrated as follows in Table 4.

It was clear that the level of blood glucose of the diabetic groups treated with vanadyl(IV) sulfate alone and vanadyl(IV) complexes (I–VI) is significantly decreased compared with the positive control group, especially Group IV_1_ that was injected by the NH_4_[(VO)(FA)(AA_1_)] complex, where the blood glucose decreased from 410 ± 15 mg/dL in the positive control group to 191 ± 4 mg/dL; so that this complex is considered the most effective one. 

In general, these results indicated that the administration of vanadyl(IV) sulfate and vanadyl(IV) complexes had moderated activity as a hypoglycemic agent, and this effect could be linked to the mimetic effect of insulin to emulate vanadyl sulfate in different tissues [50,51].

In an in vivo study, vanadyl sulfate augments glucose transport and metabolism in skeletal muscle, adipose tissue, and liver [52].

Vanadyl(IV) sulfate and vanadyl(IV) complexes administration to diabetic rats induced substantial changes in insulin hormone levels when compared with the untreated diabetic groups, especially Group IV_1_ where the insulin increased by 56%. 

Alternatively, the diabetic untreated group exhibited a significant marked decrease in the level of insulin compared with the normal control group alongside the other treated groups, where vanadyl salts can emulate many of insulin’s metabolic actions both in vitro and in vivo and enhance glycemic control with diabetes mellitus [53].

#### 3.2.2. Glutamate Pyruvate Transaminase (GPT) Enzyme Activity

Serum GPT is the major enzyme that determines liver functions, acting as an indicator of liver cell damage [54]. GPT enzyme activity in the experimental groups is illustrated in Table 4. 

It was indicated that vanadyl(IV) sulfate injection alone at a dose of 40 mg/kg body weight slightly increased the activity of GPT enzyme from 112 ± 7 in the positive control group to 124 ± 11, while the injection of vanadyl(IV) complexes at the same dose slightly decreased the serum GPT activity in the other groups. 

It is indicated that vanadyl(IV) complexes treatment had minimal side effects on liver cells of diabetic rats, according to GPT enzyme activity, in comparison with the positive control group, which shown a high increase in liver tissue damage; hence, this is a sign of the low toxicity effect of vanadyl(IV) complexes compared with vanadyl(IV) sulfate alone.

#### 3.2.3. Creatinine and Uric Acid Levels

As per the results in Table 4, diabetic rats treated with vanadyl(IV) sulfate alone and vanadyl(IV) complexes had diminished creatinine level when compared with diabetic rats, especially Group IV_3_ that was injected with the NH_4_[(VO)(FA)(AA_3_)]·H_2_O complex, which had serum creatinine decreased from 1 ± 0.2 mg/dL in the positive control group to 0.6 ± 0.2 mg/dL, and thus indicating that VO(IV) complexes had no side effect on kidneys tissue in the animal model system and greatly improved kidney functions, and stating VO(IV) complexes had an ameliorating effect on the functions of kidneys [55,56].

Uric acid increased value (hyperuricemia) observed in diabetic rats coincides with Edwards’ [57] findings that uric acid surged in diabetic mice; this may be caused by the breakage of uric acid, in case of diabetics, into substances called purines. According to our study, VO(IV) complexes decreased this value in treated diabetic rats, especially in Group IV_4_, where the uric acid decreased by 35%, and this decline in uric acid can be explained by the impaired oxidative phosphorylation processes that inhibit protein synthesis [58].

#### 3.2.4. Lactate Dehydrogenase and Glucose-6-Phosphate Dehydrogenase Activities

LDH is often used as an indicator of tissue breakdown due to its abundance in red blood cells and its functionality as an indicator of hemolysis [59]. LDH enzyme activity in the experimental groups is illustrated in Table 4. In general, serum LDH activity in STZ diabetic rats exponentially surged compared with the normal control group. VO(IV) complexes administration to STZ diabetic rats caused a significant decline in LDH activities compared with the diabetic positive control groups, especially Group IV_5_, that afforded a significant decrease in LDH by 24%. The increase of LDH activity in diabetic rats caused by LDH leakage into the blood owing to the toxicity of STZ in the liver; these results agree with who stated the same response in alloxan diabetic mice [60]. 

G6PD is the main source of the major intracellular reductant, NADPH, which is a necessity for many enzymes, including enzymes of the antioxidant pathway [61]. G6PD level decreased in all STZ diabetic rats as compared with the normal control (Table 4); such observations were reported beforehand with the exact parameters [62,63]. Diabetic rats’ treatment with vanadyl complexes increased the G6PD activity as compared with the positive diabetic groups, especially Group IV_6_, that showed a significant increase in G6PD activity.

#### 3.2.5. Levels of Hemoglobin

Table 4 illustrates the extent of hemoglobin (Hb) levels in the experimental groups. The results indicate that hemoglobin levels diminished compared with the normal control group. The reduction of hemoglobin and anemia that occur in diabetic rats mainly results from the increased RBC membrane proteins nonenzymatic glycosylation, which correlates with hyperglycemia [64]. VO(IV) complexes administration fended the increase in the level of Hb content at the end of the study compared with the untreated diabetic group. Simultaneously, the diabetic group treated with VO(IV) complexes incited a substantial surge in Hb level with reference to the normal control group, especially Group IV_1_ that afforded a significant increase in Hb by 8%, and this is an indicator of VO(IV) complexes’ low toxicity effect on the living systems of experimental animals.

#### 3.2.6. Superoxide Dismutase Enzyme (SOD)

The superoxide dismutase (SOD) is a crucial enzyme of the antioxidant system that increase their activities in the case of positive effects of an antioxidant agent in a living system. SOD scavenges the superoxide radical by transforming it into H_2_O_2_ and molecular oxygen. The SOD activity is minor in diabetes mellitus [65]; SOD diminished activity could a result of its degradation or inhibition due to increased production of free radicals. In Table 4, our results indicate the significant decrease of SOD in diabetic rats as compared with the normal control group, unlike the slight decrease in SOD in VO(IV) complexes-treated diabetic group. The treatment with VO(IV) complexes motivated the activity of SOD and may assist in controlling diabetic rats’ free radicals.

#### 3.2.7. Lipid Profile

Table 4 illustrates total cholesterol levels (TC), triglycerides (TG), HDL-c, and LDL-c. The results exhibit that total cholesterol levels (TC), triglycerides (TG), and LDL-c substantially surge in diabetic STZ rats as compared with the normal control group, while inhibition of the levels of serum HDL-c in diabetic rats was noticed, agreeing with Bolkent et al.’s [66,67] reports beforehand. The aberrant serum lipids’ high levels in diabetic animals are mainly resulting from the enhanced mobilization of free fatty acids from the peripheral deposits, as insulin hinders the hormone-sensitive lipase [68]. Extra fatty acids in diabetic mice’s serum are transformed into phospholipids and cholesterol in the liver. It is an insulin-dependent tissue that plays crucially affects glucose and lipid homeostasis and severely affected in the case of diabetes [69]. Diabetes induces a decrease in the utilization of glucose and a surge in the production of glucose in insulin-dependent tissues, e.g., liver [70]. Hypercholesterolemia’s degree is directly proportional to the severity of diabetes. This study showed that VO(IV) complexes administration significantly improved the parameters of lipid metabolism where total cholesterol (TC), triglycerides (TG), and LDL-c are substantially diminished, while HDL-c levels are significantly increased in the serum of diabetic rats. The underlying mechanism of vanadyl sulfate exertion of cholesterol’s lowering effect seems to be an inhibition in cholesterol absorption from the intestine through binding with the intestine’s bile acids and increasing bile acids excretion [71]. However, Sharma et al. [72] reported the decreasing effect of vanadyl sulfate on the cholesterol biosynthesis, especially the 3-hydroxy-3-methylglutaryl-CoA (HMG-CoA) reductase activity, a key enzyme of cholesterol biosynthesis, and/or the reducing effect that NADPH requires for fatty acids and cholesterol biosynthesis. Additionally, vanadyl may enhance hypercholesterolemia by altering lipoprotein metabolism: enhancing uptake of LDL by increasing LDL receptors [73], and/or surging the activity of lecithin cholesterol acyl transferase [74], which play a role in blood lipid regulation.

#### 3.2.8. Histopathology of Pancreas

**Figure 7:**
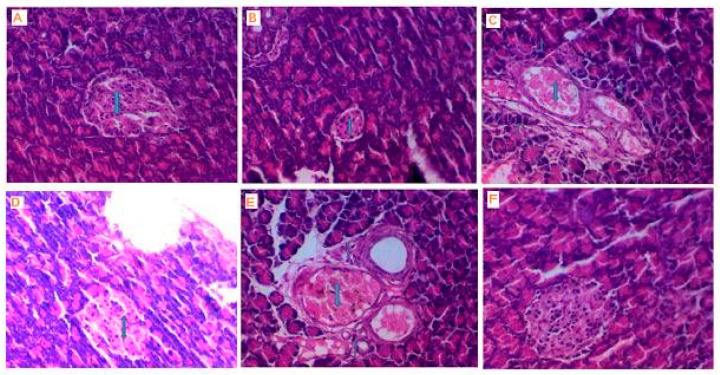
**(A)** Normal control of the pancreas. Photomicrograph of normal pancreatic tissue showing normal-sized islet of Langerhans (↕) surrounded by normal pancreatic acini. Hematoxylin and eosin (H&E) stain ×400). (**B**) Positive diabetes mellitus (DM) control of the pancreas. Photomicrograph of pancreatic tissue of diabetic rat showing atrophy of the islet of Langerhans (↕) surrounded by normal pancreatic acini. (H&E stain ×400). (**C**) Positive DM control of the pancreas. Photomicrograph of pancreatic tissue of diabetic rat showing dilated congested vascular spaces (↕) surrounded by aggregates of inflammatory cells (↑) and pancreatic acini. (H&E stain ×400). (**D**) Pancreas of the treated group with VOSO_4_. Photomicrograph of pancreatic tissue of diabetic rat treated with VOSO_4_ showing slight increase in the islet of Langerhans (↕). (H&E stain ×400). (**E**) Pancreas of the treated group with VOSO_4_. Photomicrograph of pancreatic tissue of diabetic rat treated with VOSO_4_ showing still dilated congested vascular space (↕) surrounded by few aggregates of inflammatory cells (↑). (H&E stain ×400). (**F**) Pancreas of the treated group with [(FA)(VO)(AA_1_)(NH_4_)] complex. Photomicrograph of pancreatic tissue of diabetic rat treated with NH_4_[(VO)(FA)(AA_1_)] complex, showing a good response with return of islet of Langerhans (↕) to its normal size. (H&E stain ×400).

Pancreas cells from the normal control group were all normally proportional and structured in terms of pancreatic tissue and size, as shown by the normal-sized islet of Langerhans surrounded by normal pancreatic acini. The islets consist of glucagon-secreting alpha cells and insulin-secreting beta cells, as shown in Figure 7A. On the other hand, pancreatic tissues in STZ diabetic control rats showed dilated congested vascular spaces surrounded by inflammatory cells and pancreatic acini’s aggregations. The islets are mainly inhabited by a uniform material of eosinophilic nature and few atrophic cells with reduced size. Eosinophilic materials also surround the blood vessel, as shown in Figure 7B,C. Pancreatic tissues in diabetic rats treated with only VOSO_4_ showed mild improvement of the size of the islet of Langerhans, with an expanded congested vascular space surrounded by a few inflammatory cells aggregates, as shown in Figure 7D,E. Pancreatic tissues in diabetic rats treated with folic acid/vanadyl/isoleucine system complex NH_4_[(VO)(FA)(AA_1_)] showed a good response with a return of islet of Langerhans to its normal size and absence of inflammatory cells and no eosinophilic deposits were seen, as shown in Figure 7F.

In the present study, the histopathology of the pancreas of the normal control rats yielded no notable changes in its histology across the 30-day study. On the other hand, administrating STZ incited severe pancreatic injury, decreasing the number of the islet cell and the diameter of the pancreatic islets where the islets shrunk in the diabetic rats compared with the normal ones. The islets destruction absolutely diminishes insulin, characteristic of diabetes mellitus. The administration of VOSO_4_ showed a mild islet expansion and substantially decreased pancreatic injuries within 30 days of treatment and recovered pancreatic tissue damage. The treatment of the diabetic rats with vanadyl complexes, especially NH_4_[(VO)(FA)(AA_1_)] complex, return to the normal pancreas histological structure with rich vascular supply and this may be due to the role of the prepared complexes in recovering the damage of pancreatic tissue that is caused by STZ-induced diabetes. In summary, this study assessed the STZ effect on β-cells and focused on vanadyl complexes potential in the prevention or treatment of diabetes. 

#### 3.2.9. Histopathology of Liver

**Figure 8:**
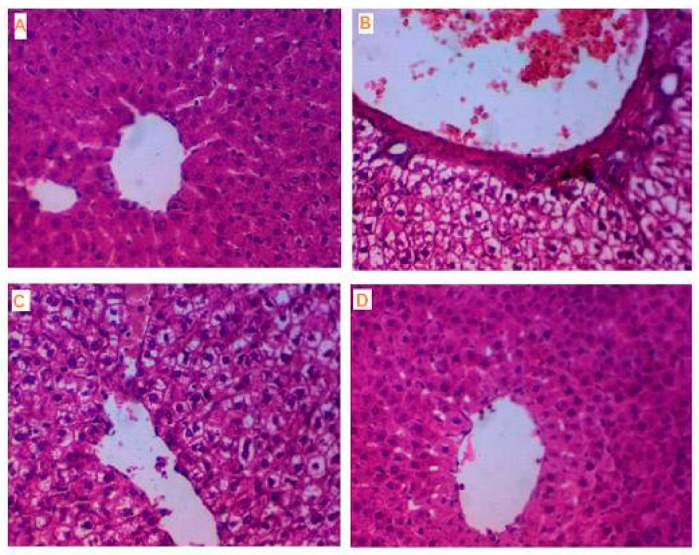
(**A**) Normal liver control. Photomicrograph of normal liver tissue showing normal size central vein (↕) surrounded by rows and cords of normal hepatocytes (↑) with central nuclei and abundant eosinophilic cytom. (H&E stain ×400). (**B**) Positive control DM Liver. Photomicrograph of liver tissue of diabetic rat showing markedly dilated congested central vein (↕) filled with red blood cells and surrounded by aggregates of inflammatory cells with rows and cords of hepatocytes showing severe fatty change (↑). (H&E stain ×400). (**C**) Liver of treated group with VOSO_4_. Photomicrograph of liver tissue from a diabetic rat treated with only VOSO_4_, showing moderately dilated congested central vein (↕) surrounded by rows and cords of hepatocytes showing moderate degree of fatty change (↑). (H&E stain ×400). (**D**) Liver of treated group with NH_4_[(VO)(FA)(AA_1_)] complex. Photomicrograph of liver tissue of diabetic rat treated with NH_4_[(VO)(FA)(AA_1_)] complex showing return to the normal state with a normal size of central vein (↕) surrounded by rows and cords of normal hepatocytes (↑). (H&E stain ×400).

From a microscopic perspective, liver samples from the normal control group showed normal structure consisting of the central vein surrounded by cords and rows of optimally healthy hepatocytes with a central nucleus and blood sinusoids, as shown in Figure 8A. Alternatively, liver tissues in the diabetic control rats group exhibited a substantial area of hepatic necrosis infiltrated with inflammatory cells with a markedly expanded congested central vein laden with red blood cells and surrounded by aggregates of inflammatory cells with rows and cords of swelled and degenerated hepatocytes with a severe fatty change, as shown in Figure 8B. Liver tissues in diabetic rats treated with only VOSO_4_ showed a mild improvement of hepatocytes with a moderately dilated congested central vein surrounded by rows and cords of hepatocytes showing a moderate degree of fatty change with normal parenchymal histology, as shown in Figure 8C. Liver tissues in diabetic rats treated with folic acid/vanadyl/isoleucine system complex NH_4_[(VO)(FA)(AA_1_)] showed good improvement of the liver tissues and returned to the normal state to the normal size of the central vein surrounded by rows and cords of normal hepatocytes and absence of inflammatory cells, as shown in Figure 8D.

In the current study, the results showed that treatment of the diabetic rats with vanadyl complexes, especially the NH_4_[(VO)(FA)(AA_1_)] complex, return the normal liver histological structure and this may be due to the role of the prepared complexes in diminishing the oxidative stress on hepatic cells and diminishing hepatocellular damage and suppression of gluconeogenesis; consequently, this may ameliorate liver damage caused by STZ-induced diabetes, which agrees with Subash et al.’s results as they studied the same effect of cinnamaldehyde on liver tissues [75]. 

## 4. Conclusions

In conclusion, the study demonstrates the synthesis of a new six intraperitoneal injections insulin-mimetic vanadyl(IV) compounds [(VO)(FA)(AA_n_)]. These compounds were synthesized by the chemical reactions between folic acid (FA), VOSO_4_, and amino acids (isoleucine, threonine, proline, phenylalanine, lysine, and glutamine) with equal molar ratio 1:1:1 in neutralized media. The characteristics of these compounds were discussed based on spectroscopic techniques such as infrared, electron spin resonance (ESR), and solid reflectance spectroscopes. An elemental analysis (XRD) study suggested the crystalline nature of the complexes; the transmission electron microscope (TEM) technique was used to investigate the surface morphology. These six newly synthesized complexes were tested in rats using a 30-day STZ-induced diabetic model. In this in vivo study, the insulin and blood glucose levels, the lipid profiles, and the histology of the pancreas and liver of the animals are qualified factors to identify the efficiency of these complexes as an alternative antidiabetic drug model. 

## Figures and Tables

**Table 1:** Elemental analysis and physical data of NH_4_[(VO)(FA)(AA_n_)] complexes.

Complex	M. wt. g/mol	% C	% H	% N	% V	*μ_eff_* *BM*	Λ_m_ (Ω^−1^cm^2^ mol^−1^)
		(calcd.)/found					
NH_4_[(VO)(FA)(AA_1_)] (C_25_H_33_N_9_O_9_V), I	654	(45.87) 45.81	(5.04) 4.98	(19.26) 19.11	(7.79) 7.71	2.1	19
NH_4_[(VO)(FA)(AA_2_)] (C_23_H_29_N_9_O_10_V), II	642	(42.99) 42.45	(4.51) 4.42	(19.62) 19.44	(7.94) 7.82	1.8	13
NH_4_[(VO)(FA)(AA_3_)]·H_2_O (C_24_H_31_N_9_O_10_V), III	656	(43.90) 43.28	(4.72) 4.66	(19.20) 19.04	(7.77) 7.72	1.7	24
NH_4_[(VO)(FA)(AA_4_)]·6H_2_O (C_28_H_43_N_9_O_15_V), IV	796	(42.21) 42.10	(5.40) 5.23	(15.82) 15.76	(6.40) 6.36	2.1	17
NH_4_[(VO)(FA)(AA_5_)] (C_25_H_34_N_10_O_9_V), V	669	(44.84) 44.39	(5.08) 5.04	(20.92) 20.83	(7.62) 7.54	2.2	21
NH_4_[(VO)(FA)(AA_6_)]·H_2_O (C_24_H_32_N_10_O_11_V), VI	687	(41.92) 41.76	(4.65) 4.59	(20.37) 20.26	(7.42) 7.31	2.2	23

**Table 2:** IR frequencies (cm^−1^) of NH_4_[(VO)(FA)(AA_n_)] complexes.

Compound	ν(O-H) ν(N-H)	ν(N-H) NH_3_	ν(C=O) COOH	ν(COO^−^)		δ(NH_3_)	δ(NH_2_)	ν(V=O)	ν(M-O)	ν(M-N)
				Asym	Sym					
FAH_2_	3539 3417 3325	--	1694	--	--	--	1607	--	--	--
AA1	--	3060	--	1582	1463	1513	--	--	--	--
AA2	--	3170	--	1631	1420	1474	--	--	--	--
AA3	--	3064	--	1624	1413	1490	--	--	--	--
AA4	--	3150	--	1626	1415	1502	--	--	--	--
AA5	--	3049	--	1582	1412	1517	--	--	--	--
AA6	--	3178	--	1633	1416	1484	--	--	--	--
I	3384 3191	--	1688	1512	1409	--	1609	1108	617 535	445
II	3387 3187	--	1686	1483	1409	--	1613	1114	617 565	450
III	3390 3250 3202	--	1687	1509	1408	--	1610	1108	616 517	440
IV	3398 3250 3195	--	1688	1505	1408	--	1613	1112	616 522	460
V	3389 3253	--	1687	1509	1407	--	1610	1112	617 517	455
VI	3400 3199	--	1687	1506	1410	--	1610	1106	617 513	452

**Table 3:** XRD diffraction patterns of the solid NH_4_[(VO)(FA)(AA_1_)] complex.

FAH_2_	AA1	Vanadium Metal
14.996, 16.870, 18.185, 20.620, 21.548, 22.862, 25.664, 28.293, and 36.738°	12.735, 19.113, 32.235, and 35.79°	38.612°

**Table 4:** Effect of vanadyl(IV) complexes (I–VI) on insulin hormone, blood glucose level, serum GPT enzyme, creatinine, uric acid level, lactate dehydrogenase, glucose-6-phosphate dehydrogenase activities, hemoglobin levels, superoxide dismutase enzyme (SOD), and lipid profile in normal and diabetic rats.

Biological Test	Group I	Group II	Group III	Group IV_1_	Group IV_2_	Group IV_3_	Group IV_4_	Group IV_5_	Group IV_6_
Insulin (IU/mL)	58 ± 2	24 ± 3	41 ± 1	53 ± 1	48 ± 2	48 ± 2	49 ± 2	47 ± 1	48 ± 2
Glucose (mg/dL)	78 ± 5	410 ± 15	284 ± 9	191 ± 4	247 ± 6	249 ± 5	245 ± 5	225 ± 4	240 ± 5
GPT (U/L)	72 ± 7	112 ± 7	124 ± 11	90 ± 5	93 ± 6	91 ± 6	91 ± 4	90 ± 5	85 ± 4
Creatinine (mg/dL)	0.5 ± 0.1	1 ± 0.2	0.9 ± 0.2	0.6 ± 0.1	0.6 ± 0.2	0.6 ± 0.2	0.6 ± 0.2	0.6 ± 0.1	0.7 ± 0.2
Uric Acid (mg/dL)	4 ± 0.2	5 ± 0.4	4 ± 0.3	4 ± 0.3	4 ± 0.3	4 ± 0.3	4 ± 0.2	4 ± 0.2	4 ± 0.2
LDH (U/L)	295 ± 15	410 ± 13	435 ± 20	355 ± 18	340 ± 22	359 ± 20	356 ± 26	330 ± 25	367 ± 19
G6PD (U/L)	12 ± 0.6	8 ± 0.5	9 ± 0.4	11 ± 0.6	11 ± 0.3	11 ± 0.4	11 ± 0.4	11 ± 0.4	11 ± 0.5
hemoglobin (gldL)	13 ± 0.4	10 ± 0.4	11 ± 0.5	14 ± 0.7	14 ± 0.5	14 ± 0.4	13 ± 0.6	14 ± 0.6	14 ± 0.5
SOD (U/mL)	308 ± 15	259 ± 22	280 ± 19	297 ± 25	290 ± 16	300 ± 20	297 ± 18	295 ± 22	302 ± 19
Cholesterol (mg/dL)	76 ± 8	211 ± 10.6	130 ± 9	102 ± 8	106 ± 5	101 ± 5	105 ± 7	104 ± 4	108 ± 5
Triglyceride (mg/dL)	140 ± 9	197 ± 12	157 ± 11	145 ± 8	149 ± 8	143 ± 9	145 ± 8	143 ± 7	143 ± 8
HDL-c (mg/dL)	42 ± 3	21 ± 2	32 ± 2	35 ± 3	35 ± 2	36 ± 3	37 ± 2	36 ± 3	36 ± 3
LDL-c (mg/dL)	31 ± 4	53 ± 5	43 ± 5	38 ± 4	42 ± 5	39 ± 4	39 ± 5	39 ± 4	38 ± 4
